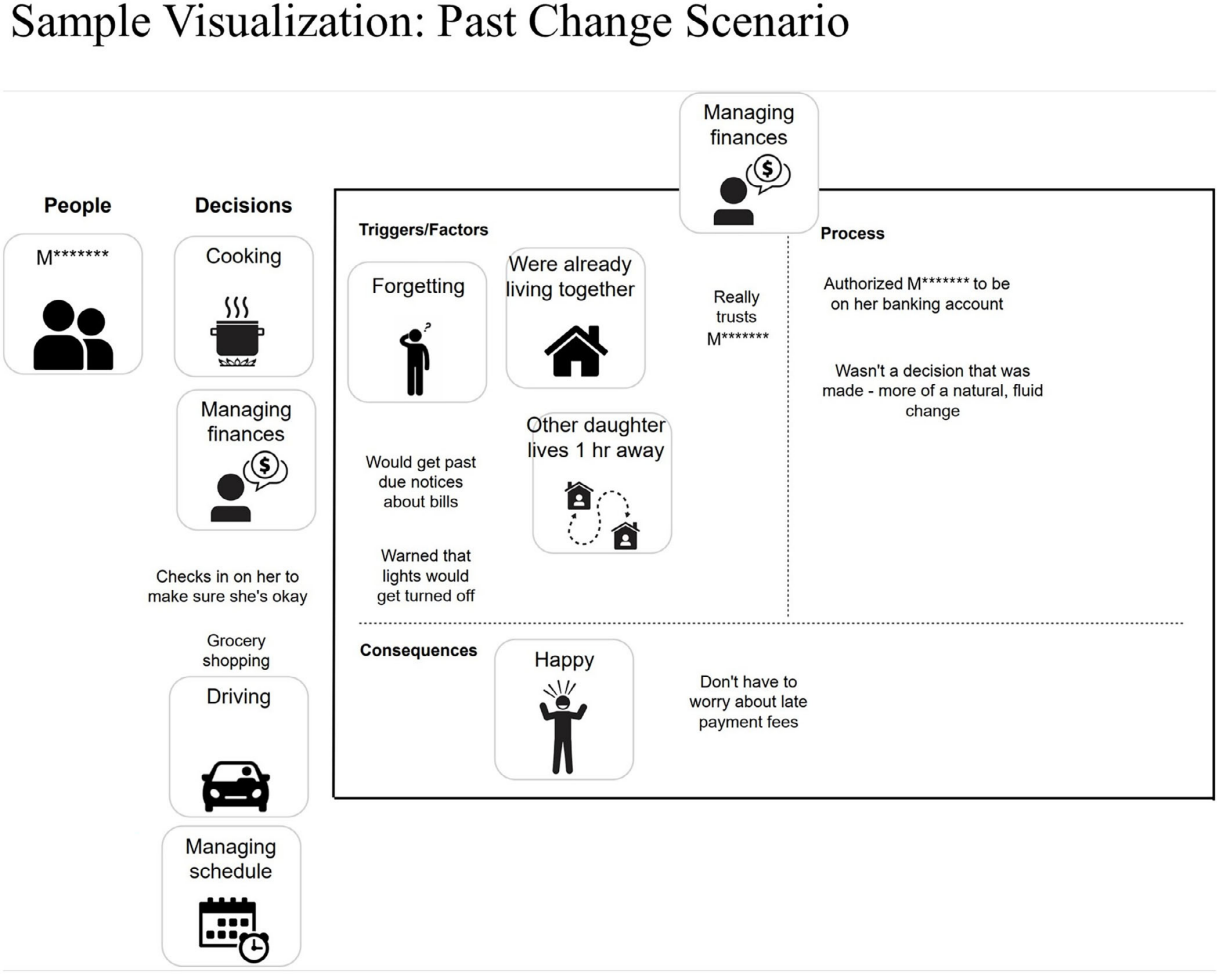# Facilitating Conversations about Supportive Care Options in the Context of Cognitive Impairment using an Online Visual Elicitation Tool

**DOI:** 10.1002/alz.087167

**Published:** 2025-01-09

**Authors:** Anne M Turner, Annie T. Chen, Claire E. Child, Lyndsey M. Miller

**Affiliations:** ^1^ University of Washington, Seattle, WA USA; ^2^ Oregon Health & Science University, Portland, OR USA; ^3^ Oregon Center for Aging & Technology (ORCATECH), Portland, OR USA

## Abstract

**Background:**

Persons with cognitive impairment may experience difficulties with language and cognition that interfere with their ability to make and communicate decisions. We developed an online visual tool to facilitate conversations about their preferences concerning supportive care.

**Methods:**

We conducted Zoom interviews with persons with mild cognitive impairment (MCI) and mild to moderate dementia, using storytelling and a virtual tool designed to facilitate discussion. Each interview sought to discuss decision‐making in the context of three scenarios, one concerning a past decision about supportive care, and two hypothetical scenarios based on decreasing abilities and increasing care needs. Interviewers shared their screen with the visual tool, which used the visual diagramming platform draw.io (https://www.drawio.com/) to create an online “canvas.” Interviewers could drag icons (representing people, places, safety, emotions, activities, and issues) or type text onto the canvas to communicate concepts. We developed the tool with a multidisciplinary team of designers and researchers, and with input from people with cognitive impairment. Key user‐centered design goals for the tool included focusing on simplicity, minimizing cognitive load, and using concepts and images that resonated with participants. We also used pilot interviews to refine interviewers’ techniques for eliciting preferences with the tool. We examined the utility of the tool for engaging participants in dialogic interactions with the interviewer, and the cognitive and communicative processes exhibited by participants.

**Results:**

We conducted fifteen interviews with persons with MCI, and mild or moderate dementia [mean age 77.8 years ± 7.1 (SD), 9 male, 6 female]. With respect to dialogic interactions, interviewers actively used the visualization technique to facilitate the conversation; participants engaged with the interface to varying degrees. Common communicative issues included participants being unresponsive to the question or providing unclear responses. During the sessions participants also exhibited uncertainty, introspection, and self‐awareness.

**Conclusion:**

We present a visual technique to engage persons with cognitive impairment in dialogue about complex decisions. We hope to explore the potential use of this technique in research, clinical, and community settings.